# Role of Support Oxygen Vacancies in the Gas Phase Hydrogenation of Furfural over Gold

**DOI:** 10.1007/s10562-017-2228-9

**Published:** 2017-10-23

**Authors:** Maoshuai Li, Laura Collado, Fernando Cárdenas-Lizana, Mark A. Keane

**Affiliations:** Chemical Engineering, School of Engineering and Physical Sciences, Heriot-Watt University, Edinburgh, EH14 4AS Scotland, UK

**Keywords:** Selective hydrogenation, Furfural, Furfuryl alcohol, Oxygen vacancies, Supported Au

## Abstract

**Abstract:**

We have examined the role of support oxygen vacancies in the gas phase hydrogenation of furfural over Au/TiO_2_ and Au/CeO_2_ prepared by deposition–precipitation. Both catalysts exhibited a similar Au particle size distribution (1–6 nm) and mean (2.8–3.2 nm). Excess H_2_ consumption during TPR is indicative of partial support reduction, which was confirmed by O_2_ titration. Gold on CeO_2_ with a higher redox potential exhibited a greater oxygen vacancy density. A lower furfural turnover frequency (*TOF*) was recorded over Au/CeO_2_ than Au/TiO_2_ and is linked to suppressed H_2_ chemisorption capacity and strong –C=O interaction at oxygen vacancies that inhibited activity. Gold on non-reducible Al_2_O_3_ as benchmark exhibited greater H_2_ uptake and delivered the highest furfural *TOF*. Full selectivity to the target furfuryl alcohol was achieved over Au/TiO_2_ and Au/Al_2_O_3_ at 413 K and over Au/CeO_2_ at 473 K with hydrogenolysis to 2-methylfuran at higher reaction temperature (523 K). A surface reaction mechanism is proposed to account for the activity/selectivity response.

**Graphical Abstract:**

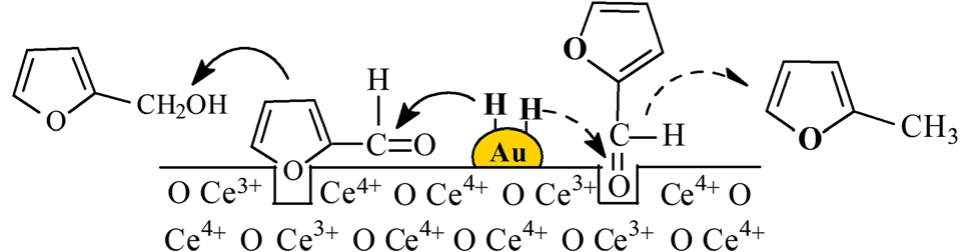

## Introduction

Oxygen vacancies in metal oxides (e.g. titanium, cerium, iron and vanadium oxides) are defects generated by the loss of lattice oxygen as a result of high temperature (≥ 673 K) annealing in ultra-high vacuum [1], chemical reduction (by H_2_ or CO) [2] and/or electron irradiation [3]. The formation and properties of these vacancies have been the subject of theoretical (DFT) and experimental (UPS, XPS, EELS, IR, EPR, STM) work [4–6]. Contributions due to oxygen vacancies have been established in catalytic water–gas shift [7], steam reforming of oxygenates [8], CO oxidation [9] and hydrodeoxygenation [10]. Moreover, the presence of these defects can modify the electronic characteristics (via electron transfer) [11], particle size (by stabilisation at vacancy sites) [12] and chemical properties (metal-support interaction) [13] of the supported metal phase (Pt [7], Ag [11], Au [13] and Pd [14]), which impact on reactant adsorption/activation. Hydrogenation is a key process in the food, petrochemical, pharmaceutical and agrochemical sectors [15]. The effect of surface oxygen vacancies in catalytic hydrogenation is still a subject of debate. Enhanced activity and (–C=O reduction) selectivity reported for Pt/CeO_2_ [16] and Au/Fe_2_O_3_ [17] in the hydrogenation of crotonaldehyde and benzalacetone was attributed to facilitated activation of the carbonyl function at oxygen vacancies and/or electron-rich metal nano-particles. On the other hand, a (threefold) decrease in crotonaldehyde hydrogenation activity was observed following incorporation of CeO_2_ (by impregnation with Ce(NO_3_)_3_) on Ru/Al_2_O_3_ and ascribed to strong –C=O interaction with oxygen deficient sites [18]. Tian et al. [19] studying the hydrogenation of cinnamaldehyde over Au/CeO_2_ suggested a preferential –C=C– adsorption on Au^δ+^ (resulting from electron transfer to support defects) to explain lower selectivity in terms of –C=O reduction. In the hydrogenation of *p*-chloronitrobenzene, unwanted hydrodechlorination was reported for Au/Ce_0.62_Zr_0.38_O_2_ and ascribed to –C–Cl scission at vacancy sites [20]. In this study, we consider the role of oxygen vacancies (on reducible TiO_2_ and CeO_2_) in determining the catalytic performance of supported Au in gas phase hydrogenation of –C=O using furfural as model reactant. Gold on non-reducible alumina serves as a benchmark catalyst.

## Experimental

### Materials and Catalyst Preparation

Commercial TiO_2_ (P25, Degussa) and CeO_2_ (Sigma-Aldrich) were used as received. The supported Au catalysts were prepared by deposition–precipitation using urea (100-fold excess, Riedel-de Haën, 99%) with HAuCl_4_ (1.5 × 10^− 3^−3.0 × 10^− 3^ M, 400 cm^3^, Sigma-Aldrich, 99%). A suspension containing the oxide carrier (10 g) was heated to 353 K (2 K min^− 1^) where the pH progressively increased to *ca*. 7 after 3–4 h as a result of urea decomposition [21]. The solid obtained was separated by filtration, washed with distilled water until chlorine free (from AgNO_3_ test) and dried in He (45 cm^3^ min^− 1^) at 373 K (2 K min^− 1^) for 5 h. The resultant sample was sieved (ATM fine test sieves) to mean particle diameter = 75 μm, activated at 2 K min^− 1^ to 523 K in 60 cm^3^ min^− 1^ H_2_, cooled to ambient temperature and passivated in 1% v/v O_2_/He for 1 h for ex situ characterisation. Synthesis and activation of the benchmark Au/Al_2_O_3_ catalyst is described in detail elsewhere [22].

### Catalyst Characterisation

The Au content was measured by atomic absorption spectroscopy (Shimadzu AA-6650 spectrometer with an air-acetylene flame) from the diluted extract in aqua regia (25% v/v HNO_3_/HCl). Temperature programmed reduction (TPR), H_2_ and O_2_ chemisorption measurements were conducted on the CHEM-BET 3000 (Quantachrome Instrument) unit with data acquisition/manipulation using the TPR Win^™^ software. Samples were loaded into a U-shaped Pyrex glass cell (3.76 mm i.d.) and heated in 17 cm^3^ min^− 1^ (Brooks mass flow controlled) 5% v/v H_2_/N_2_ to 523 K at 2 K min^− 1^. The effluent gas passed through a liquid N_2_ trap and H_2_ consumption was monitored by a thermal conductivity detector (TCD). The activated samples were swept with 65 cm^3^ min^− 1^ N_2_ for 1.5 h, cooled to 413 K and subjected to H_2_ chemisorption by pulse (10 μl) titration. In blank tests, there was no measurable H_2_ uptake on the oxide supports alone. Oxygen chemisorption post-TPR was employed to determine the extent of support reduction [23], where the samples were reduced as described above, swept with 65 cm^3^ min^− 1^ He for 1.5 h, cooled to 413 K with pulse (50 μl) O_2_ titration. It has been demonstrated previously that Au contribution to total O_2_ adsorbed is negligible [24]. Nitrogen physisorption was performed using the commercial Micromeritics Gemini 2390p system. Samples were outgassed at 423 K for 1 h prior to analysis. Total specific surface area (SSA) was calculated using the standard single point BET method. X-ray diffractograms (XRD) were recorded on a Bruker/Siemens D500 incident X-ray diffractometer using Cu Kα radiation, scanning at 0.02° per step over the range 20° ≤ 2θ ≤ 80°. The diffractograms were identified against the JCPDS-ICDD reference standards, i.e. Au (04-0784), anatase-TiO_2_ (21-1272), rutile-TiO_2_ (21-1276), CeO_2_ (43-1002) and Ce_2_O_3_ (23-1048). Gold particle morphology was examined by scanning transmission electron microscopy (STEM, JEOL 2200FS field emission gun-equipped unit), employing Gatan Digital Micrograph 1.82 for data acquisition/manipulation. Samples for analysis were prepared by dispersion in acetone and deposited on a holey carbon/Cu grid (300 Mesh). The surface area weighted mean Au particle size (*d*) was based on a count of at least 300 particles, according to

1 $${d_{}}=\frac{{\sum\limits_{i} {{n_i}d_{i}^{3}} }}{{\sum\limits_{i} {{n_i}d_{i}^{2}} }}$$

where *n*_*i*_ is the number of particles of diameter *d*_*i*_.

### Catalyst Testing

Hydrogenation of furfural (Sigma-Aldrich, 99%) was carried out at atmospheric pressure and 413–523 K in situ after activation in a continuous flow fixed bed tubular reactor (15 mm i.d.). Reactions were conducted under operating conditions that ensured negligible mass/heat transport limitations. A layer of borosilicate glass beads served as preheating zone, ensuring that the furfural reactant was vaporised and reached reaction temperature before contacting the catalyst. Isothermal conditions (± 1 K) were ensured by diluting the catalyst bed with ground glass (75 µm), which was mixed thoroughly with catalyst before loading into the reactor. Reaction temperature was continuously monitored by a thermocouple inserted in a thermowell within the catalyst bed. Furfural was delivered as *n*-butanolic (Sigma-Aldrich, > 99%) solutions to the reactor *via* a glass/Teflon air-tight syringe and Teflon line using a microprocessor controlled infusion pump (Model 100 kd Scientific) at a fixed calibrated flow rate. A co-current flow of furfural and H_2_ was adjusted to *GHSV* = 1 × 10^4^ h^− 1^. The molar Au to inlet reactant molar feed rate (*n*/*F*) spanned the range 4 × 10^− 3^–30 × 10^− 3^ h. Passage of furfural in a stream of H_2_ through the empty reactor or over support alone did not result in any detectable conversion. The reactor effluent was condensed in a liquid N_2_ trap for subsequent analysis using a Perkin-Elmer Auto System XL gas chromatograph equipped with a programmed split/splitless injector and a flame ionisation detector, employing a DB-1 (50 m × 0.33 mm i.d., 0.20 μm film thickness) capillary column (J&W Scientific). Data acquisition and manipulation were performed using the TurboChrom Workstation Version 6.3.2 (for Windows) chromatography data system. Furfuryl alcohol and 2-methylfuran were used as supplied (Sigma-Aldrich, 99%) for product identification/analysis. All gases (O_2_, H_2_, N_2_ and He) were of high purity (BOC, > 99.98%). Furfural fractional conversion (*X*) is defined by

2 $$X=\frac{{[furfural{\text{]in}} - [furfural]{\text{out}}}}{{[furfural]{\text{in}}}}$$

and selectivity (*S*) to product (j) is given by

3 $$S_{\text{ j }}(\% )=\frac{{[product]{\text{j, out}}}}{{[furfural]{\text{in}} - [furfural]{\text{out}}}} \times 100$$

where the subscripts “in” and “out” refer to the inlet and outlet gas streams. Turnover frequency (*TOF*, rate per active site) was calculated using Au dispersion measurements from STEM as described elsewhere [25]. Repeated reactions with different samples from the same batch of catalyst delivered raw data reproducibility and carbon mass balances that were within ± 5%.

## Results and Discussion

### Catalyst Characterisation

**Fig. 1:**
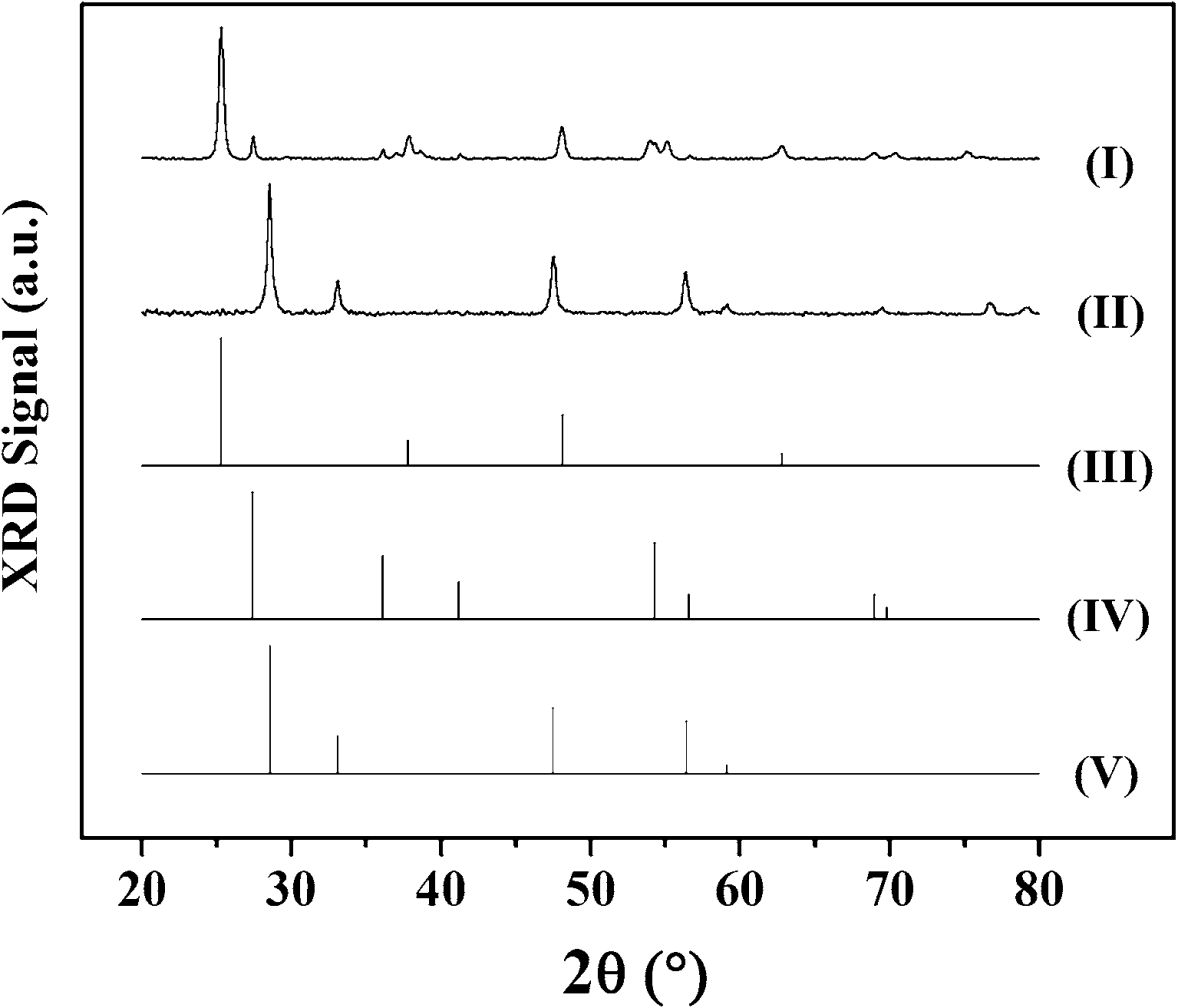
XRD patterns for (*I*) Au/TiO_2_ and (*II*) Au/CeO_2_ with JCPDS-ICDD reference diffractograms for (*III*) anatase-TiO_2_ (21-1272), (*IV*) rutile-TiO_2_ (21-1276) and (*V*) CeO_2_ (43-1002)

**Fig. 2:**
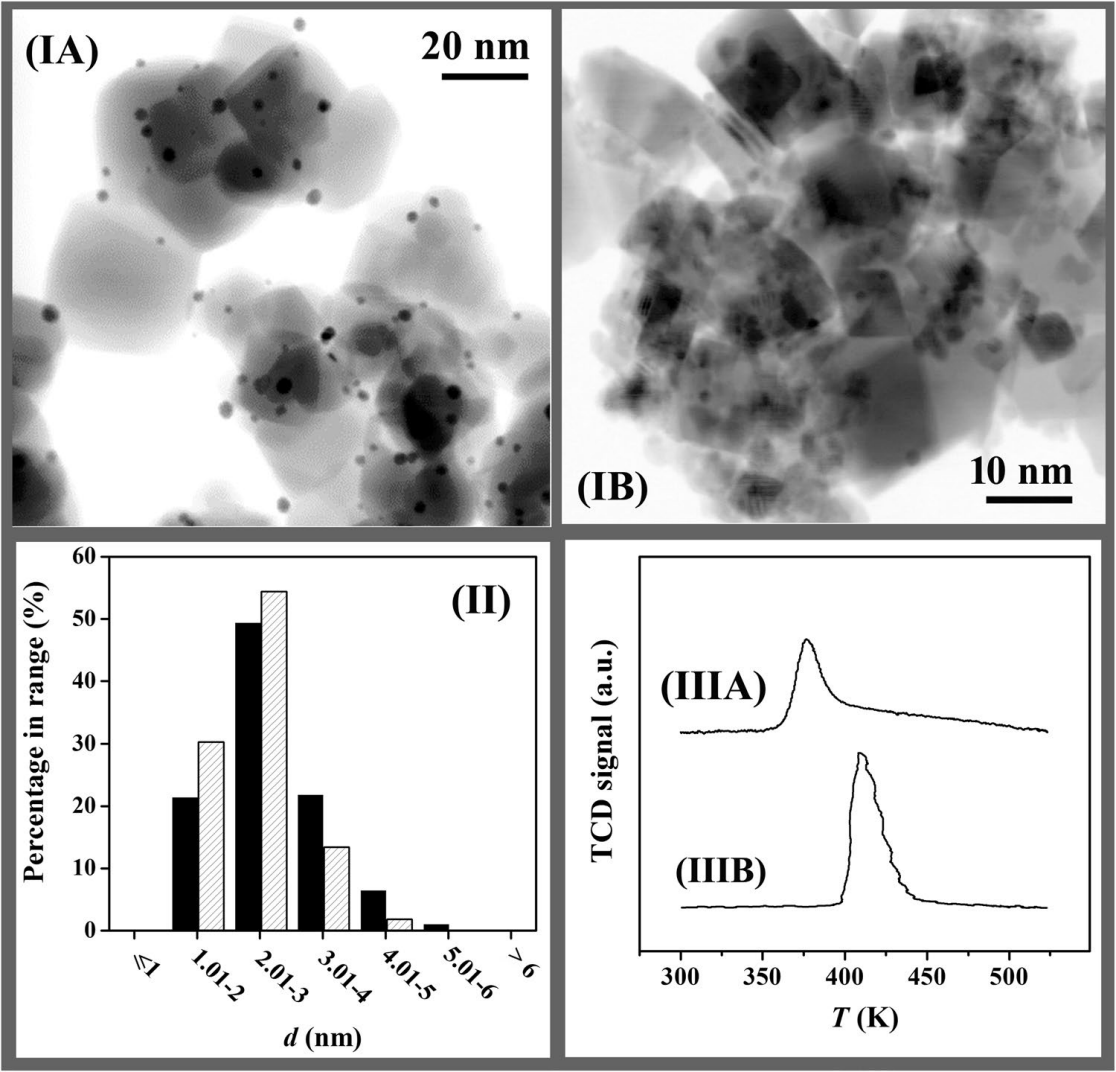
**(I)** Representative STEM images with **(II)** associated Au particle size distribution histograms and **(III)** temperature programmed reduction (TPR) profiles for **A** Au/TiO_2_ (solid bars) and **B** Au/CeO_2_ (hatched bars)

The physicochemical characteristics of Au/TiO_2_ and Au/CeO_2_ are given in Table 1; the values for Au/Al_2_O_3_ are taken from a prior publication [22]. The samples contained a similar Au loading (0.6–0.8 mol%) where the SSA match values reported for TiO_2_ (50 m^2^ g^− 1^) [26] and CeO_2_ (36–67 m^2^ g^− 1^) [11] supported group IB metal catalysts. XRD analysis (Fig. 1) of Au/TiO_2_ (I) revealed a mixture of tetragonal anatase [2θ = 25.3°, 37.8°, 48.1° and 62.8°, (III)] and rutile [2θ = 27.4°, 36.1°, 41.2°, 54.3°, 56.6°, 69.0° and 69.8°, (IV)] phases with an anatase : rutile ratio (5:1) consistent with Degussa P25 [27]. The XRD pattern of Au/CeO_2_ (II) presents principal peaks (at 2θ = 28.6°, 33.1°, 47.5°, 56.4° and 59.1°) characteristic of CeO_2_ (V). In both cases, there were no diffraction peaks due to Au (principal peak 2θ = 38.1°; JCPDS-ICDD card 04-0784), diagnostic of a well dispersed (< 5 nm) metal phase [24]. This was confirmed by STEM analysis (Fig. 2) where both samples exhibited quasi-spherical Au nanoparticles (IA, IB) with similar size range (1–6 nm) and mean [(II); Table 1]. The TPR profile of Au/TiO_2_ (Fig. 2IIIA) shows a single peak (*T*_*max*_ = 376 K) with an associated H_2_ consumption that exceeded the amount required for the formation of Au^0^ (Table 1) but far lower than that (6200 μmol g^− 1^) required for Ti^4+^ → Ti^3+^. This suggests a partial reduction of the support, notably at the Au-support interface [28]. Reduction of Au/CeO_2_ (Fig. 2IIIB) exhibited H_2_ consumption at higher *T*_*max*_ (418 K). Liu and Yang [29] reported a dependency of Au^3+^ reducibility on support redox properties where weaker interactions with TiO_2_ compared with CeO_2_ rendered the Au^3+^ component more susceptible to reduction. In the TPR of Au/CeO_2_, H_2_ consumed was greater than the requirements for Au precursor reduction but considerably less than bulk Ce^4+^ → Ce^3+^ transformation (2900 μmol g^− 1^). There were no signals due to Ce_2_O_3_ (main peak 2θ = 29.5°; JCPDS-ICDD card 23-1048) in the XRD pattern. Increased H_2_ uptake during activation of Au/CeO_2_ relative to Au/TiO_2_ suggests a greater degree of support reduction. This agrees with the higher redox potential [30] of CeO_2_ (*E*_redox_, Table 1). In contrast, TPR analysis of benchmark Al_2_O_3_ (with the lowest *E*_redox_) supported Au (*d* = 4.3 nm) generated an equivalent H_2_ consumption to the theoretical value for Au^3+^ → Au^0^, confirming support non-reducibility (Table 1).

**Table 1:** Gold loading, specific surface area (SSA), mean Au particle size from STEM analysis (*d*), H_2_ consumption during TPR, H_2_ and O_2_ uptake and support standard redox potential (*E*_redox_) for the supported Au catalysts

Catalyst	Au/TiO_2_	Au/CeO_2_	Au/Al_2_O_3_^a^
Au loading (mol%)	0.8	0.7	0.6
SSA (m^2^ g^− 1^)	52	64	166
*d* (nm)	3.2	2.8	4.3
TPR H_2_ consumption (µmol g^− 1^)	174^b^/147^c^	495^b^/61^c^	87^b^/84^c^
H_2_ chemisorption (µmol g_Au_^−1^)^d^	146	87	318
*E*_redox_ (V)^e^	− 0.6	1.6	− 1.7
O_2_ chemisorption (µmol g^− 1^)^d^	8	90	1

^a^Data from [22]

^b^Experimental measurements

^c^H_2_ required for Au^3+^ → Au^0^

^d^Measured at 413 K

^e^Taken from [30]

The number of surface oxygen vacancies can be quantified by oxygen titration [23, 31]. Oxygen chemisorption post-TPR was employed to determine the extent of support reduction; the values are given in Table 1. Decreasing O_2_ uptake (Au/CeO_2_ > Au/TiO_2_ > Au/Al_2_O_3_) matched the sequence of decreasing support redox potential and H_2_ consumption during TPR. Oxygen vacancy formation in TiO_2_ has been established by *in situ* EPR following reduction (in H_2_) over 573–1073 K [32]. Boccuzzi et al. [33] using FTIR spectroscopy demonstrated H_2_ dissociation on Au sites supported on TiO_2_ (reduced at 523 K) with spillover that resulted in surface reduction. It has been established (by DFT calculation and STM) that bare ceria surfaces can be reduced (Ce^4+^ → Ce^3+^) to generate oxygen defects post-activation in H_2_ at 400–900 K [34, 35]. Addition of Au to ceria facilitates support reduction (273–573 K) during TPR [36]. The performance of supported Au catalysts in hydrogenation is determined by the capacity for H_2_ adsorption/dissociation [24]. Hydrogen chemisorption (at 413 K, Table 1) on Au/TiO_2_ was measurably higher than Au/CeO_2_. Gold particle size and support interactions impact on H_2_ adsorption [37–39]. Corner and edge sites associated with smaller Au particles (< 10 nm) have been identified as active for H_2_ dissociation [37]. Mean Au size is close for the three catalysts (Table 1). The order of decreasing H_2_ uptake (Au/Al_2_O_3_ > Au/TiO_2_ > Au/CeO_2_) matches that of increasing O_2_ chemisorption (Table 1). Lower uptake on Au/TiO_2_ and Au/CeO_2_ can be linked to metal encapsulation due to surface Au diffusion into the bulk (573–673 K) that is facilitated by oxygen vacancies on reducible oxides [40, 41], an effect more pronounced for Au/CeO_2_ with higher vacancy density.

The characterisation results demonstrate the generation of nano-scale Au particles on TiO_2_ and CeO_2_ with a greater density of surface oxygen vacancies and lower H_2_ uptake on Au/CeO_2_. Gold on non-reducible Al_2_O_3_ with similar metal size is a suitable candidate to evaluate the effect of oxygen vacancies on furfural hydrogenation over supported Au.

### Catalytic Response

**Fig. 3:**
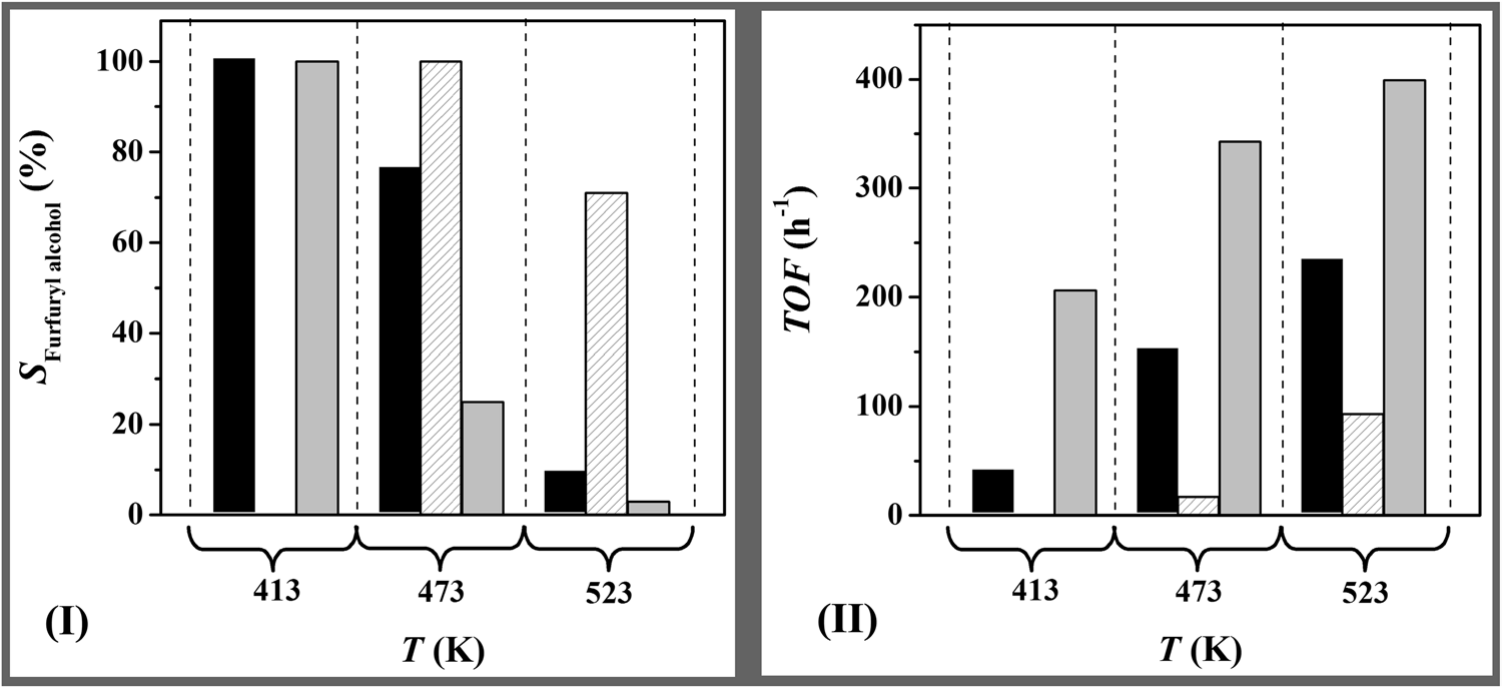
Variation of **I** furfuryl alcohol selectivity (*S*_Furfuryl alcohol_) at an equivalent fractional furfural conversion and **II** turnover frequency (*TOF*) with temperature for reaction over Au/TiO_2_ (solid bars), Au/CeO_2_ (hatched bars) and Au/Al_2_O_3_ (grey bars). *Reaction conditions: P* = 1 atm; *T* = 413–523 K

A search through the literature did not produce any reported application of TiO_2_ or CeO_2_ supported Au catalysts in furfural hydrogenation. We can flag the work of Ohyama et al. [42] on high pressure (38 atm) liquid phase hydrogenation of 2-hydroxylmethyl-5-furfural where reaction over Au/TiO_2_ resulted in furan ring opening and Au/CeO_2_ promoted carbonyl group reduction at a (tenfold) lower activity. No explanation was given for the observed differences in selectivity or activity. Gas phase furfural hydrogenation at 413 K over Au/TiO_2_ generated the target furfuryl alcohol as sole product (Fig. 3I). Reaction over Au/Al_2_O_3_ delivered an appreciably higher selective turnover frequency (*TOF*) and Au/CeO_2_ was inactive (Fig. 3II). This activity response can be linked to differences in H_2_ chemisorption capacity (Table 1, in the order Au/Al_2_O_3_ > Au/TiO_2_ > Au/CeO_2_) under reaction conditions. Zanella et al. [43] identified H_2_ dissociation as rate-determining in the chemoselective hydrogenation of aldehydes over supported Au. In this study, the *TOF* normalised with respect to H_2_ chemisorption capacity was lower for Au on reducible supports (notably Au/CeO_2_) relative to Au/Al_2_O_3_. This suggests a contribution due to furfural adsorption at surface oxygen vacancies. These vacancies can act as sites for strong binding of oxygenated reactants [34, 44]. The higher density of oxygen vacancies on Au/CeO_2_ (Table 1) can act to stabilise surface adsorbed furfural, resulting in lower reaction rates. The action of oxygen vacancies to inhibit –C=O reduction is in line with the lower activity recorded for cinnamaldehyde hydrogenation (to cinnamyl alcohol) over Au/CeO_2_ relative to Au/MgO-Al_2_O_3_ reported by Tian et al. [19] though this possibility was not proposed by the authors. An increase in temperature (≥ 473 K) (i) elevated *TOF* where Au/CeO_2_ consistently delivered lower rates (Fig. 3II) and (ii) resulted in a switch in selectivity from furfuryl alcohol to 2-methylfuran. Reaction over Au/TiO_2_ and Au/Al_2_O_3_ at 523 K generated 2-methylfuran as principal product (*S* > 91%). In the case of Au/CeO_2_, a higher reaction temperature (473 K) resulted in the selective transformation of furfural to furfuryl alcohol while a further increase (to 523 K) generated 2-methylfuran as by-product. These results suggest that elevated temperatures favour activation of –C=O for hydrogenolytic cleavage, which finds agreement in results reported for Cu/MgO [45].

**Fig. 4:**
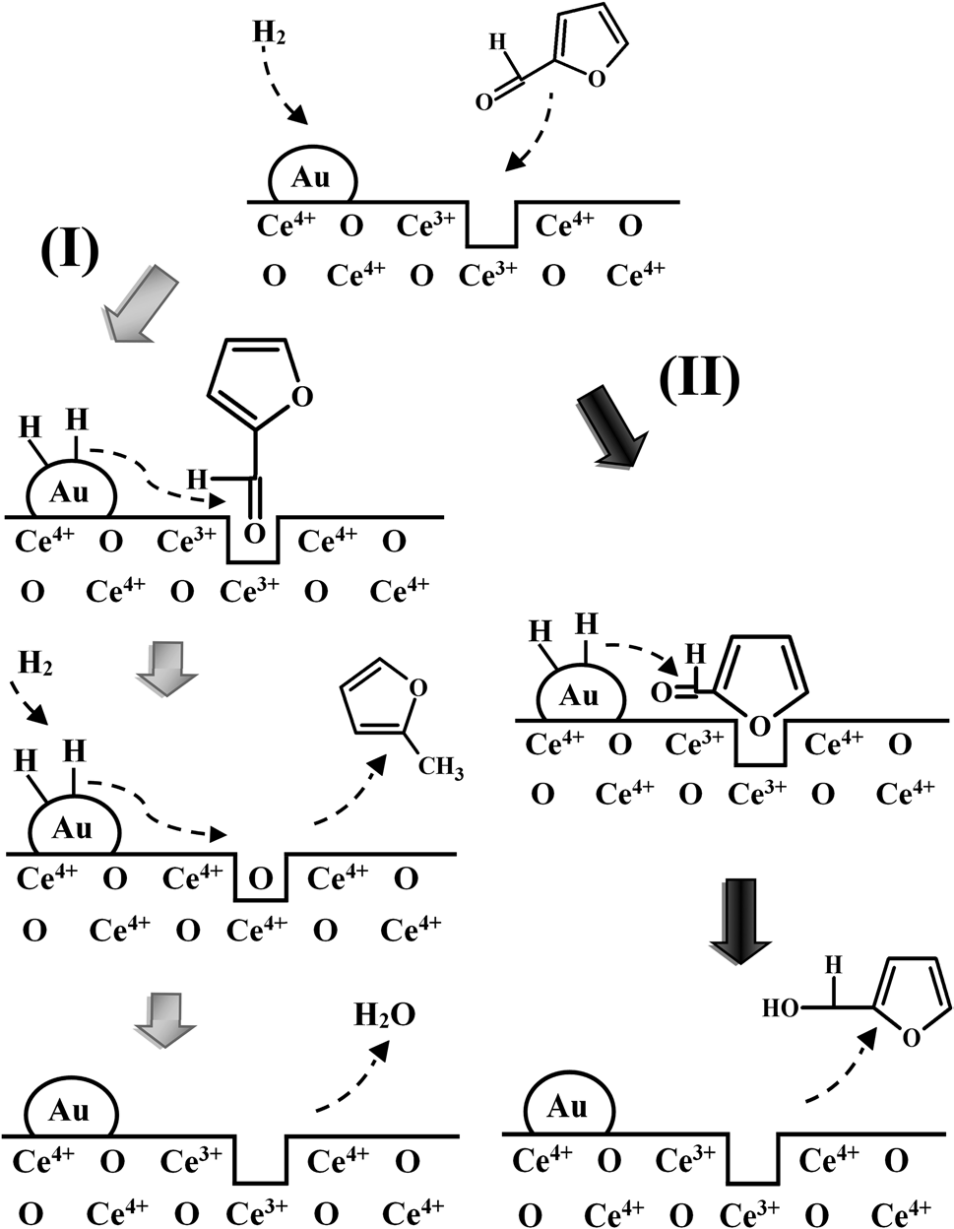
Proposed surface furfural adsorption/activation and reaction for Au on reducible supports (CeO_2_) at oxygen vacancies via (*I*) the carbonyl group (grey arrows) or (*II*) furan ring (black arrow*s*)

Ceria supported Au with a greater oxygen vacancy density exhibited a distinct catalytic response compared with Au/TiO_2_ and Au/Al_2_O_3_. We propose a reaction mechanism that involves direct participation of surface vacancies where the carbonyl group of furfural can be ‘‘anchored’’ to a vacancy (Ce^3+^) site (see Fig. 4I), forming a covalent Ce–O bond with a high energy of interaction [46] that stabilises the surface reactant and lowers reactivity. The (stabilised) carbonyl group can be activated for reaction at higher temperature (523 K) where hydrogenolysis to 2-methylfuran results from hydrogen scission of –C=O. The surface Ce^3+^ sites are oxidised by the abstracted oxygen from the carbonyl group. Oxygen vacancies can be regenerated by H_2_ dissociated on Au sites that spills over to the support, resulting in a continuous creation/consumption/regeneration of these vacancies. Another possible adsorption mode is through the furan ring oxygen that interacts with the electron-rich vacancy site [47] (Fig. 4II). The energy barrier for reaction is lower relative to the covalent –C=O ‘‘anchoring’’ at vacancies. In this case, the carbonyl group is attacked by reactive hydrogen to form the target furfuryl alcohol with subsequent desorption. Oxygen defects are also present on Au/TiO_2_ but at a lower density with a consequent higher conversion to furfuryl alcohol at lower reaction temperature. Interaction of –C=O with Lewis acid sites (Al^3+^) on non-reducible Al_2_O_3_ facilitates –C=O activation [27] and results in greater reactivity and higher *TOF*.

## Conclusion

We have established structure sensitivity in the gas phase hydrogenation of furfural over (0.7–0.8 mol%) Au/TiO_2_ and Au/CeO_2_ (mean Au particle size = 2.8–3.2 nm). A surface reaction mechanism is proposed to explain the role of surface oxygen vacancies in determining hydrogenation activity and selectivity. Reaction over Au/CeO_2_ delivered lower furfural *TOF*, which can be linked to inhibited H_2_ chemisorption capacity. The greater oxygen vacancy density on CeO_2_ (with higher redox potential) post-TPR served to stabilise the –C=O function and lower reactivity. Full selectivity to the alcohol was achieved over Au/TiO_2_ (at 413 K) and Au/CeO_2_ (at 473 K) where hydrogenolysis to 2-methylfuran was promoted at 523 K. Reaction over Au on non-reducible Al_2_O_3_ delivered higher furfural *TOF* (at 413 K) to furfuryl alcohol with 2-methylfuran formation at *T* ≥ 473 K.
